# Associations Among School Absenteeism, Gastrointestinal and Respiratory Illness, and Income — United States, 2010–2016

**DOI:** 10.15585/mmwr.mm6809a1

**Published:** 2019-03-08

**Authors:** David Berendes, Ashley Andujar, Lisa C. Barrios, Vincent Hill

**Affiliations:** ^1^Division of Foodborne, Waterborne, and Environmental Diseases, CDC; ^2^Division of Adolescent and School Health, CDC.

Control of communicable diseases in children, including respiratory and diarrheal illnesses that affect U.S. school-aged children, might require public health preventive efforts both in the home and at school, a primary setting for transmission. National Health Interview Survey (NHIS) data on school absenteeism and gastrointestinal illness in the United States during 2010–2016 were analyzed to identify associations among income, illness, and absenteeism. Prevalence of gastrointestinal and respiratory illnesses in the 2 weeks preceding the survey increased as income decreased. Although the likelihood of missing any school days during the past year decreased with reduced income, among children missing school, those from low-income households missed more days of school than did children from higher income households. Although the reason for absenteeism cannot be ascertained from this analysis, these data underscore the importance of preventive measures, such as hand hygiene promotion and education, and the opportunity for both homes and schools to serve as an important point for implementation of public health preventive measures, including hand hygiene practice and education.

Data from the 2010–2016 NHIS (*1*) were analyzed. NHIS is an annual, national survey on household and child health in the noninstitutionalized U.S. population, administered continually throughout the year. Estimates based on these data are designed to meet National Center for Health Statistics standards (standard errors ≤0.3) (*1*). Family income data were linked to information about the school-aged child (5–17 years) with regard to 1) any school absenteeism in the last year, 2) number of days absent, and 3) gastrointestinal illness or respiratory illness (occurrence of a cold) during the 2 weeks preceding the interview. Income was assessed using NHIS-computed income brackets and by annual federal poverty level* thresholds computed by the U.S. Census Bureau (by family size). The statistical software R (version 3.4.3, R Foundation for Statistical Computing) was used to compare school absenteeism, illness, and income using linear and logistic regression models, unadjusted and adjusted for age and sex of the child and year of survey. P-values <0.05 were considered statistically significant.

A total of 645,209 respondents provided income information, and 61,482 (9.6%) were selected to provide data about their school-age child’s health and days of school missed. Respondents varied across income categories, with 31% earning <$35,000 per year and 19% below the federal poverty level (Table 1). Sixty-nine percent of children missed ≥1 day of school the previous year, and approximately 15% missed ≥6 days (mean = 3.3 days per child). In the 2 weeks preceding the survey, prevalences of gastrointestinal and respiratory illnesses were 5% and 13%, respectively.

**TABLE 1:** Number and percentage of respondents reporting school absences among children aged 5–17 years, by federal poverty level (FPL) status, income, school absence, and gastrointestinal and respiratory illnesses — National Health Interview Survey, 2010–2016

Characteristic	No. of respondents (%)							
	Year							
	2010	2011	2012	2013	2014	2015	2016	Total
Below FPL*	1,540 (19.6)	1,748 (19.6)	1,860 (19.9)	1,783 (19.5)	1,895 (19.8)	1,570 (18.0)	1,164 (14.7)	11,560 (18.8)
**Annual income**								
<$34,999	2,643 (33.6)	3,001 (33.6)	3,179 (34.0)	2,979 (32.7)	2,919 (30.6)	2,466 (28.2)	1,914 (24.1)	19,101 (31.1)
$35,000–$49,999	1,056 (13.4)	1,252 (14.0)	1,190 (12.7)	1,216 (13.3)	1,145 (12.0)	984 (11.3)	790 (10.0)	7,633 (12.4)
$50,000–$74,999	1,300 (16.5)	1,424 (16.0)	1,493 (16.0)	1,430 (15.7)	1,396 (14.6)	1,328 (15.2)	1,158 (14.6)	9,529 (15.5)
$75,000–$99,999	879 (11.2)	979 (11.0)	1,124 (12.0)	1,039 (11.4)	1,092 (11.4)	916 (10.5)	953 (12.0)	6,982 (11.4)
≥$100,000	1,991 (25.3)	2,263 (25.4)	2,366 (34.0)	2,460 (27.0)	2,999 (31.4)	3,039 (34.8)	3,119 (39.3)	18,237 (30.0)
**School days absent during previous year**								
0	2,275 (28.9)	2,722 (30.5)	3,230 (34.5)	2,849 (31.2)	3,099 (32.4)	2,700 (30.9)	2,410 (30.4)	19,285 (31.4)
Any	5,594 (71.1)	6,197 (69.5)	6,122 (65.5)	6,275 (68.8)	6,452 (67.6)	6,033 (69.1)	5,524 (69.6)	42,197 (68.6)
1–2	2,150 (27.3)	2,524 (28.3)	2,725 (29.1)	2,627 (28.8)	2,779 (29.1)	2,553 (29.2)	2,364 (29.8)	17,722 (28.8)
3–5	2,136 (27.1)	2,365 (26.5)	2,207 (23.6)	2,353 (25.8)	2,421 (25.3)	2,157 (24.7)	2,005 (25.3)	15,644 (25.4)
6–10	857 (10.9)	874 (9.8)	811 (8.7)	866 (9.5)	866 (9.1)	900 (10.3)	788 (9.9)	5,962 (9.7)
≥11	451 (5.7)	434 (4.9)	379 (4.1)	429 (4.7)	386 (4.0)	423 (4.8)	367 (4.6)	2,869 (4.7)
Mean days absent (SD)	3.65 (7.30)	3.36 (7.10)	2.95 (6.02)	3.29 (6.37)	3.07 (6.31)	3.40 (6.88)	3.32 (6.64)	3.28 (6.66)
**Illness during past 2 weeks**								
Gastrointestinal	413 (5.3)	470 (5.3)	399 (4.3)	437 (4.8)	476 (5.0)	392 (4.5)	371 (4.7)	2,958 (4.8)
Respiratory	1,041 (13.2)	1,255 (14.1)	995 (10.6)	1,299 (14.2)	1,210 (12.7)	1,111 (12.7)	997 (12.6)	7,908 (12.9)

**Abbreviation: **SD = standard deviation.

* FPL represents an indicator used to define the boundary for those eligible for federal aid; FPL is defined by the U.S. Department of Health and Human Services annually each January to adjust for inflation and is proportional to the size of the household.

Reported school absence during the previous school year and reported respiratory or gastrointestinal illness during the previous 2 weeks were categorized by household income (Table 2). Compared with children in each of the other income categories, children in the lowest income bracket households (earning <$35,000 per year) had lower likelihood of missing school during the previous year (65% versus 67%–73%) and higher prevalence of gastrointestinal illness (6% versus 4%–5%) and respiratory illness (14% versus 12%–13%) in the previous 2 weeks. Adjusting for age, sex, and year of survey, children in the lowest income bracket were 4%–12% less likely to miss school (95% confidence interval [CI] = 1%–16%), but 12%–28% more likely to have had a recent gastrointestinal illness (95% CI = 2%–35%). Children in the lowest income bracket were also 6%–11% more likely to have had a respiratory illness, although comparisons with each of the next two highest income brackets ($35,000–$49,999 and $50,000–$74,999) were not statistically different.

**TABLE 2:** Number and percentage of respondents reporting school absence and illness among children aged 5–17 years, by income and federal poverty level (FPL) status — National Health Interview Survey, 2010–2016

Characteristic	No. of respondents (%)						
	Income					Poverty status*	
	<$35,000	$35,000–$49,999	$50,000–$74,999	$75,000–$99,999	≥$100,000	Below FPL	At or above FPL
**School days absent**							
0	6,710 (35.1)	2,497 (32.7)	2,831 (29.7)	1,906 (27.3)	5,341 (29.3)	4,108 (35.5)	13,781 (29.7)
Any	12,391 (64.9)	5,136 (67.3)	6,698 (70.3)	5,076 (72.7)	12,896 (70.7)	7,452 (64.5)	32,546 (70.3)
PR (95% CI)	Referent	1.04 (1.00 to 1.07)	1.08 (1.05 to 1.12)	1.12 (1.08 to 1.16)	1.09 (1.0 to 1.12)	Referent	1.09 (1.0 to 1.12)
aPR^†^ (95% CI)	Referent	1.04 (1.00–1.07)	1.08 (1.05–1.12)	1.12 (1.09–1.16)	1.09 (1.07–1.12)	Referent	1.09 (1.07–1.12)
1–2	4,499 (23.6)	2,065 (27.1)	2,814 (29.5)	2,203 (31.6)	6,141 (33.7)	2,640 (22.8)	14,077 (30.4)
3–5	4,562 (23.9)	1,919 (25.1)	2,512 (26.4)	1,955 (28.0)	4,696 (25.7)	2,767 (23.9)	12,071 (26.1)
6–10	2,079 (10.9)	752 (9.9)	978 (10.3)	674 (9.7)	1,479 (8.1)	1,259 (10.9)	4,443 (9.6)
≥11	1,251 (6.5)	400 (5.2)	394 (4.1)	244 (3.5)	580 (3.2)	786 (6.8)	1,955 (4.2)
Mean (SD) all	3.72 (7.99)	3.42 (6.96)	3.16 (5.95)	3.07 (4.75)	2.90 (5.89)	3.80 (8.34)	3.20 (6.22)
Est^§^ (95% CI)	Referent	-0.30 (-0.48 to -0.12)	-0.56 (-0.72 to -0.39)	-0.65 (-0.83 to -0.47)	-0.82 (-0.96 to -0.69)	Referent	-0.60 (-0.74 to -0.47)
aEst^†^ (95% CI)	Referent	-0.32 (-0.50 to -0.15)	-0.58 (-0.74 to -0.42)	-0.67 (-0.86 to -0.49)	-0.87 (-1.00 to -0.73)	Referent	-0.65 (-0.78 to -0.51)
Mean (SD)^¶^	5.74 (9.32)	5.08 (7.98)	4.50 (6.66)	4.22 (5.12)	4.10 (6.64)	5.90 (9.77)	4.55 (7.00)
Est (95% CI)	Referent	-0.65 (-0.90 to -0.41)	-1.23 (-1.46 to -1.01)	-1.52 (-1.76 to -1.27)	-1.63 (-1.82 to -1.45)	Referent	-1.35 (-1.54 to -1.16)
aEst^†^ (95% CI)	Referent	-0.68 (-0.93 to -0.44)	-1.27 (-1.50 to -1.05)	-1.56 (-1.81 to -1.32)	-1.71 (-1.90 to -1.53)	Referent	-1.41 (-1.60 to -1.22)
**Illness during past 2 weeks**							
Gastrointestinal	1,086 (5.7)	359 (4.7)	475 (5.0)	309 (4.4)	729 (4.0)	689 (6.0)	2129 (4.6)
PR (95% CI)	Referent	0.83 (0.7 to 0.93)	0.88 (0.7 to 0.98)	0.79 (0.6 to 0.88)	0.70 (0.6 to 0.77)	Referent	0.77 (0.7 to 0.84)
aPR^†^ (95% CI)	Referent	0.83 (0.7 to 0.94)	0.88 (0.7 to 0.98)	0.79 (0.6 to 0.89)	0.72 (0.6 to 0.79)	Referent	0.78 (0.7 to 0.85)
Respiratory	2,625 (13.7)	979 (12.8)	1,222 (12.8)	847 (12.1)	2,235 (12.3)	1,596 (13.8)	5,919 (12.8)
PR (95% CI)	Referent	0.93 (0.8 to 1.00)	0.93 (0.8 to 1.00)	0.88 (0.8 to 0.95)	0.89 (0.8 to 0.94)	Referent	0.93 (0.8 to 0.98)
aPR^†^ (95% CI)	Referent	0.94 (0.8 to 1.01)	0.94 (0.8 to 1.01)	0.89 (0.8 to 0.96)	0.91 (0.8 to 0.96)	Referent	0.94 (0.8 to 0.99)

**Abbreviations:** aEst = adjusted estimate (from linear regression); aPR = adjusted prevalence ratio; CI = confidence interval; Est = estimate (from linear regression); PR = prevalence ratio; SD = standard deviation.

* FPL represents an indicator used to define the boundary for those eligible for federal aid; FPL is defined by the U.S. Department of Health and Human Services annually each January to adjust for inflation and is proportional to the size of the household. Because the poverty line data includes both income and number of household members, there were more missing values for poverty level; therefore, the numbers in the below FPL and at or above FPL groups do not sum to the number in all income groups.

^†^ Adjusted for age and sex of child, as well as year of data collection.

^§^ Estimated difference from reference.

^¶^ Among those missing ≥1 school day only.

Results were similar when comparing children living below the federal poverty level with those at or above it. Children living below the poverty level were significantly less likely to have missed school during the past year (65% versus 70%), and also significantly more likely to have had a gastrointestinal illness (6% versus 5%) or respiratory illness (14% versus 13%) in the preceding 2 weeks (Table 2). Specifically, children living below the poverty level were 9% less likely to have missed a day of school during the last year (95% CI = 6%–12%), but were 22% more likely to have had a gastrointestinal illness (95% CI = 15%–28%) and 6% more likely to have had a respiratory illness (95% CI = 1%–11%) during the 2 weeks preceding the survey.

Among children whose parents reported respiratory or gastrointestinal illness during the preceding 2 weeks, the percentage who missed any school during the last year increased with increasing income level. Among children who had gastrointestinal illness, 84.6% (family income <$35,000), 86.1% ($35,000–$49,999), 90.3% ($50,000–$74,999), 89.6% ($75,000–$99,999), and 87.4% (≥$100,000) missed school in the past year. Similarly, 83.7% of children living below the poverty level with gastrointestinal illness missed school, compared with 88.3% of those living at or above the poverty level. Among children in the household income brackets listed above who had a respiratory illness during the preceding 2 weeks, 78.5%, 79.7%, 80.5%, 82.3%, and 81.3%, respectively, missed school, and 77.6% of children living in households below the federal poverty level missed school compared with 81.2% of those living at or above the poverty level. Differences for both gastrointestinal and respiratory illnesses were significant in bivariable analyses (e.g., chi-square tests), but not in final model risk ratios.

When analyzed by the number of days missed, children in the lowest income bracket (<$35,000) missed a mean of 0.3–0.9 more days in the last year compared with children in other income brackets (Table 2). Among only children who missed ≥1 school day, the differences were larger (mean = 0.7–1.7 more days). Similarly, overall, children living below the federal poverty level missed an average of 0.6 more days of school per year than did children in higher income households; among only those who missed ≥1 day of school, the difference increased to 1.4 days.

## Discussion

Compared with children from higher income households, those from lower income households were more likely to have had a gastrointestinal or respiratory illness during the 2 weeks preceding the survey. Although children from lower income households were less likely to have missed any days of school during the last year, those who did miss school missed more days than did children from higher income households.

The combination of increased illness prevalence and absenteeism with decreasing income status highlights the need for accessible, affordable resources and interventions at home and school. Multiple barriers faced by children in low-income households could explain these findings, including lack of access to preventive health care (*2*). Although targeted social distancing, such as a requirement for absence from school might be an effective recommended course of action to protect public health (*3*,*4*), low-income parents might not have the opportunities (e.g., paid sick leave from work) to be able to implement this. These circumstances might affect both their children’s ability to stay home from school and health-seeking behaviors (*5*). In the long-term, longer periods of absenteeism could be associated with adverse educational outcomes (*6*).

The findings in this report are subject to at least two limitations. First, although NHIS collects health and school absence data generalizable to the U.S. population as a whole, the reasons for school absence are not collected. Second, both health and school absence data are self-reported, making them subject to recall bias, and the data are not consistent in their respective recall timelines (preceding 2 weeks versus preceding year). However, recall of self-reported illness and school absenteeism is likely to be more accurate for the recent past (*7*); thus the association between reporting of recent illness and school absenteeism is likely to be strengthened. In addition, subgroup differences in illness, though small (one percentage point) fell outside of the survey margins of error.

From a public health perspective, these findings highlight a need for resources for, and attention to, preventive measures to keep children in school. Beyond practices in the home, schools have opportunities to serve as settings for preventing transmission of communicable diseases. Some school-based programs promoting handwashing, and more generally hand hygiene, have been found to be effective in reducing gastrointestinal and respiratory illnesses and associated absenteeism (*8*). Research suggests that peer support and provision of soap can increase handwashing and reduce absenteeism related to both gastrointestinal and respiratory illnesses (*9*). However, further study of sustained, community-based encouragement of proper hand hygiene practices as effective, low-cost means of preventing such illnesses is needed. Ongoing health promotion activities in schools can increase awareness and understanding of handwashing with soap as an effective and affordable way to prevent transmission of infectious diseases. Increased public awareness of the importance of hand hygiene, as promoted by Global Handwashing Day (observed each year on October 15), is important to promoting public health and reducing the transmission of illness.

**Summary**

**What is already known about this topic?**

Gastrointestinal and respiratory infections are important illnesses that affect U.S. school-aged children. Schools can serve as primary settings of transmission.

**What is added by this report?**

During 2010–2016, parents of children from low-income households were more likely to report recent childhood gastrointestinal and respiratory illnesses than were higher income parents. Although parents of children from low-income households were less likely to report missing any school, these children tended to miss more school days, on average, when they did miss school.

**What are the implications for public health practice?**

Public health partners could expand prevention efforts to decrease transmission of gastrointestinal and respiratory illnesses, especially low-cost measures such as promoting hand hygiene education in schools.

## References

[1] National Center for Health Statistics, CDC. NHIS—National Health Interview Survey. Atlanta, GA: US Department of Health and Human Services, CDC; 2019. https://www.cdc.gov/nchs/nhis/index.htm

[2] Morsy L, Rothstein R. Five social disadvantages that depress student performance: why schools alone can’t close achievement gaps. Washington, DC: Economic Policy Institute, 2015 https://www.epi.org/files/pdf/86987.pdf

[3] Glass RJ, Glass LM, Beyeler WE, Min HJ. Targeted social distancing design for pandemic influenza. Emerg Infect Dis 2006;12:1671–81. 10.3201/eid1211.060255 17283616 PMC3372334

[4] Qualls N, Levitt A, Kanade N, ; CDC Community Mitigation Guidelines Work Group. Community mitigation guidelines to prevent pandemic influenza—United States, 2017. MMWR Recomm Rep 2017;66(No. RR-1). 10.15585/mmwr.rr6601a1 28426646 PMC5837128

[5] DeRigne L, Stoddard-Dare P, Quinn L. Workers without paid sick leave less likely to take time off for illness or injury compared to those with paid sick leave. Health Aff (Millwood) 2016;35:520–7. 10.1377/hlthaff.2015.0965 26953308

[6] Balfanz R, Byrnes V. The importance of being in school: a report on absenteeism in the nation’s public schools. Baltimore, MD: Johns Hopkins University Center for Social Organization of Schools; 2012. http://new.every1graduates.org/wp-content/uploads/2012/05/FINALChronicAbsenteeismReport_May16.pdf

[7] Arnold BF, Galiani S, Ram PK, Optimal recall period for caregiver-reported illness in risk factor and intervention studies: a multicountry study. Am J Epidemiol 2013;177:361–70. 10.1093/aje/kws281 23364878

[8] Wang Z, Lapinski M, Quilliam E, Jaykus LA, Fraser A. The effect of hand-hygiene interventions on infectious disease-associated absenteeism in elementary schools: a systematic literature review. Am J Infect Control 2017;45:682–9. 10.1016/j.ajic.2017.01.018 28242074

[9] Bowen A, Ma H, Ou J, A cluster-randomized controlled trial evaluating the effect of a handwashing-promotion program in Chinese primary schools. Am J Trop Med Hyg 2007;76:1166–73. 10.4269/ajtmh.2007.76.1166 17556631

