# Correction and Republication: Associations Among School Absenteeism, Gastrointestinal and Respiratory Illness, and Income—United States, 2010 – 2016

**DOI:** 10.15585/mmwr.mm6902a5

**Published:** 2020-01-17

On March 8, 2019, *MMWR* published “Associations Among School Absenteeism, Gastrointestinal and Respiratory Illness, and Income—United States, 2010-2016” (*1*). On March 18, 2019, the National Center for Health Statistics, which manages the National Health Interview Survey, notified the authors of a concern that the analysis had not correctly accounted for the complex survey design and did not use the imputed income files or the survey weights. *MMWR* was informed about these concerns on March 27, 2019. The authors have corrected these errors and confirmed that they do not change the interpretation or the conclusions of the original report. In addition, several wording changes were made to clarify the text and interpretation. In accordance with December 2017 guidance from the International Committee of Medical Journal Editors (*2*), *MMWR* is republishing the report (*3*). The republished report includes the original report with clearly marked corrections in supplementary materials.
